# AbOmpA in *Acinetobacter baumannii*: exploring virulence mechanisms of outer membrane-integrated and outer membrane vesicle-associated AbOmpA and developing anti-infective agents targeting AbOmpA

**DOI:** 10.1186/s12929-025-01147-5

**Published:** 2025-05-27

**Authors:** Man Hwan Oh, Md Minarul Islam, Nayeong Kim, Chul Hee Choi, Minsang Shin, Woo Shik Shin, Je Chul Lee

**Affiliations:** 1 Department of Microbiology, Dankook University, Cheonan, 31116 Republic of Korea; 2 Smart Animal Bio Institute, Dankook University, Cheonan, 31116 Republic of Korea; 3 Department of Microbiology, School of Medicine, Kyungpook National University, Daegu, 41944 Republic of Korea; 4 Department of Microbiology, School of Medicine, Chungnam National University, Daejeon, 35015 Republic of Korea; 5 Untreatable Infectious Disease Institute, Kyungpook National University, Daegu, 41944 Republic of Korea; 6 Department of Pharmaceutical Sciences, Northeast Ohio Medical University, Rootstown, OH USA

**Keywords:** *Acinetobacter baumannii*, Outer membrane protein A, Outer membrane vesicle, Virulence factor, Anti-infective agent

## Abstract

*Acinetobacter baumannii* is notorious for its antimicrobial resistance and its potential to cause epidemics in hospital settings, which pose a global health threat. Although this microorganism is traditionally considered a low-virulence pathogen, extensive research has been conducted on its virulence and pathogenesis in recent years. Advances in understanding the virulence mechanisms of *A. baumannii* have prompted a shift in the development of anti-infective agents. The outer membrane protein A (AbOmpA) of *A. baumannii* is a key virulence factor both in vitro and in vivo. AbOmpA exists in three forms: outer membrane-integrated AbOmpA, outer membrane vesicle (OMV)-associated AbOmpA, and free proteins. Given that outer membrane-integrated AbOmpA has been implicated in the virulence and antimicrobial resistance of *A. baumannii*, many studies have focused on outer membrane-integrated AbOmpA as a therapeutic target for combating drug-resistant *A. baumannii*, and have led to the discovery of small molecules, polypeptides, and antimicrobial peptides targeting AbOmpA. However, the pathophysiological role of OMV-associated AbOmpA and its impact on AbOmpA-targeting agents remain unclear. This review summarizes the current knowledge of AbOmpA and critically discusses OMV-associated AbOmpA in relation to virulence and its potential impact on AbOmpA-targeted therapies to provide a better understanding of AbOmpA for the development of novel therapeutics against *A. baumannii*.

## Background

### Clinical significance of *Acinetobacter baumannii*

*Acinetobacter baumannii* has emerged as an important nosocomial pathogen, primarily affecting severely ill or immunocompromised patients [1]. This microorganism is responsible for a variety of opportunistic infections, including pneumonia, urinary tract infections, skin and soft tissue infections, meningitis, and bacteremia [1–4]. The mortality rate among patients infected with *A. baumannii*, particularly those with bloodstream infections and ventilator-associated pneumonia, ranges from 28 to 43% and 40 to 70%, respectively [5, 6]. The high mortality is attributed to both host factors and antimicrobial resistance of the bacteria [7].

*A. baumannii* is notorious for its resistance to commonly used antimicrobial agents. The emergence and spread of drug-resistant *A. baumannii* pose a substantial threat to public health [8, 9]. *A. baumannii* is a member of ‘ESKAPE’ pathogens *(Enterococcus faecium, Staphylococcus aureus, Klebsiella pneumoniae, A. baumannii, Pseudomonas aeruginosa,* and *Enterobacter* species), which are known for their ability to escape antimicrobial agents due to increasing multidrug-resistance (MDR) [10]. Moreover, carbapenem-resistant *A. baumannii* (CRAB) has been classified as a critical priority pathogen in the WHO Bacterial Priority Pathogens List of 2024 [11]. *A. baumannii* exhibits antimicrobial resistance through intrinsic resistance mechanisms and the acquisition of resistance genes via horizontal gene transfer, leading to MDR, extensively drug-resistant, and pandrug-resistant bacteria [12, 13]. Over the past 2 decades, CRAB strains have been increasingly reported worldwide. Class D β-lactamases, including *bla*_OXA-23-_, *bla*_OXA-24-_, and *bla*_OXA-58-like_ genes, are the primary resistance mechanisms of carbapenem in global epidemic clones, particularly global clone 2 (also known as international clone II) [13–20]. Additionally, class B metallo-β-lactamases, such as *bla*_IMP_, *bla*_VIM_, and *bla*_NDM_, have also been implicated in carbapenem resistance among CRAB strains [13–17, 21]. Tigecycline, a third-generation tetracycline derivative, and the older drug colistin (polymyxin E) are considered last-line defenses against CRAB, but resistance to these agents has been increasingly observed [22]. The overexpression of resistance-modulation-division (RND) efflux pumps, such as AdeABC, AdeFGH, and AdeIJK, plays a key role in tigecycline resistance of *A. baumannii* [22, 23]. Modifications of lipid A or the loss of lipooligosaccharides (LOS) confer colistin resistance in this bacterium [24–26]. Mutations in *pmrA* and *pmrB*, which encode components of the PmrAB two-component regulatory system, are responsible for lipid A modification [27]. Plasmid-mediated *mcr* genes, which encode phosphoethanolamine transferase, also contribute to colistin resistance [28]. Furthermore, resistance to new agents, such as cefiderocol (siderophore-conjugated cephalosporin), has also emerged in clinical *A. baumannii* strains, with reduced expression of the siderophore receptor gene *pirA* linked to cefiderocol resistance [29]. Given the growing antimicrobial resistance and the global expansion of drug-resistant strains, there is an urgent need to develop new therapeutics against drug-resistant *A. baumannii*. Anti-virulence strategies represent a promising alternative approach to combat drug-resistant bacteria.

### AbOmpA: a key virulence factor

*A. baumannii* has been regarded as a low-virulence pathogen; however, significant progress in understanding its virulence and pathogenesis has been made over the past 2 decades. Several virulence factors have been identified in *A. baumannii*, including outer membrane proteins (OmpA, Omp33-36, Omp22, and CarO) [30–36], capsular polysaccharides [37, 38], LOS [39–41], phospholipases [42, 43], metal acquisition systems (iron, zinc, and manganese) [44–48], serum resistance-associated factors (PBP 7/8, CipA, Tuf, and SurA1) [49–52], Csu pili [53–55], and host cell death-inducing factors (AbeD, OmpR/EnvZ, and) [56–58]. Additionally, genes such as *feoA*, *bfnL*, *basB*, *yfgC*, *hisF, mtnN*, and, *oatA* are linked to in vivo virulence of *A. baumannii* [59]. Bacterial regulatory systems, including quorum sensing system (AbaI/R), two-component regulatory systems (BfmRS, PmrAB, and GacSA), and stringent response regulatory systems (ppGpp and DksA), also play a role in regulating virulence-associated genes in *A. baumannii* [60–63]. The regulation of virulence-associated genes and the expression of virulence traits in *A. baumannii* are complex.

Among the well-characterized virulence determinants, the outer membrane protein A (AbOmpA) of *A. baumannii* stands out as a key virulence factor, contributing directly or indirectly to biofilm formation [32, 64], surface motility [65], adherence and invasion of host cells [66], outer membrane vesicle (OMV) biogenesis [67], serum resistance [68], host cell death [31, 69], autophagy [70], induction of innate immunity [71], dissemination into the blood [72], and immune modulation [73]. The overproduction of AbOmpA has been identified as an independent risk factor for the high mortality rate in patients with pneumonia and bacteremia caused by *A. baumannii* [74]. Therefore, AbOmpA represents a potential target for new anti-infective drugs against *A. baumannii*. Many studies have focused on neutralizing AbOmpA on living bacteria as a novel therapeutic strategy for combating drug-resistant *A. baumannii* infections. However, AbOmpA exists in three distinct forms—outer membrane-integrated AbOmpA, OMV-associated AbOmpA, or free proteins—during in vitro culture and in vivo infection [75, 76]. *A. baumannii* released large quantities of AbOmpA into the extracellular environment via OMVs [76]. Therefore, a comprehensive understanding of both outer membrane-integrated AbOmpA and OMV-associated AbOmpA is crucial for elucidating their roles in virulence and developing new therapeutics targeting AbOmpA.

## Structure and biophysical characteristics of AbOmpA

### Structure and function of outer membrane-integrated AbOmpA

**Fig. 1:**
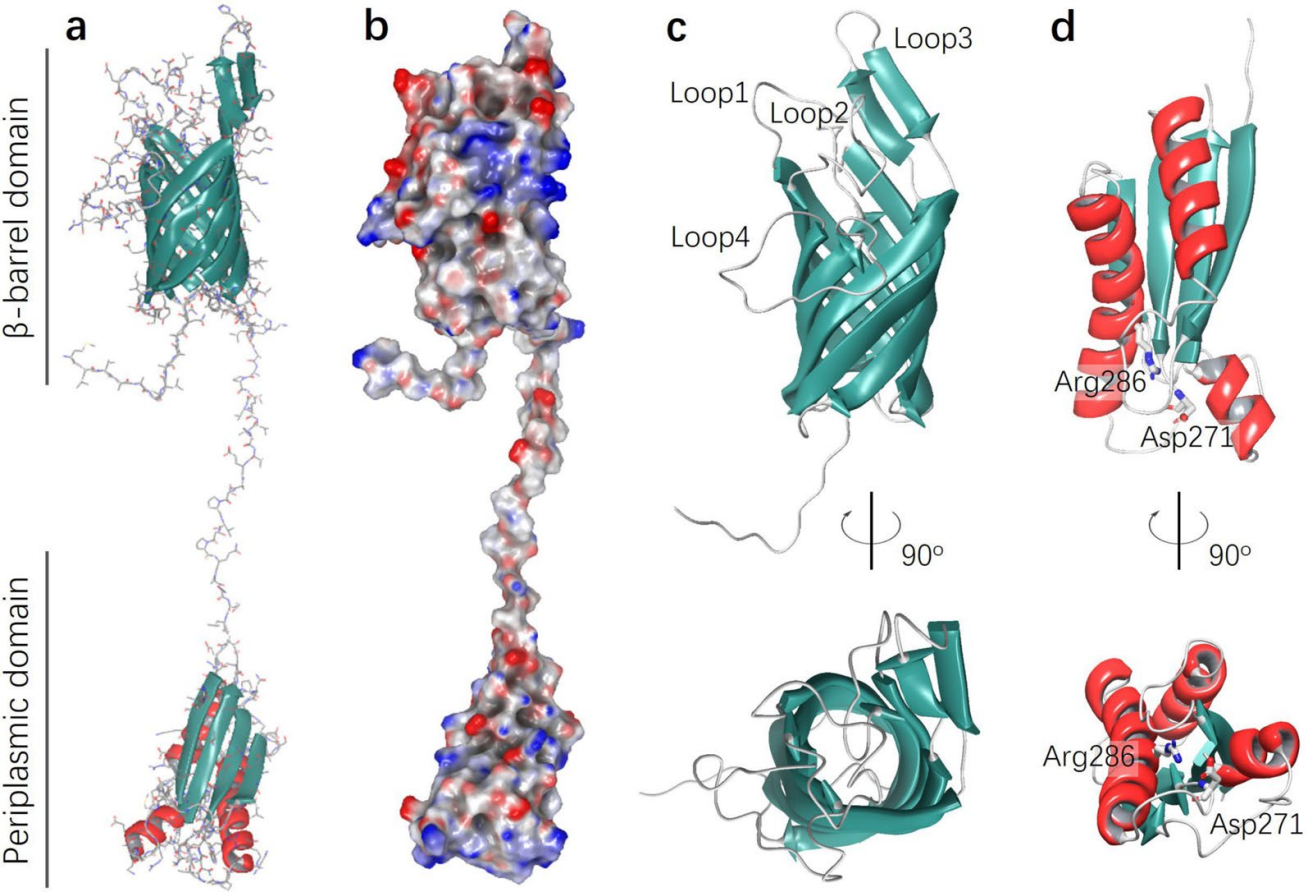
Three-dimensional structural representation of AbOmpA provides a comprehensive overview of its structural organization. **a** Ribbon diagram of the full-length homology model of AbOmpA. The N-terminal β-barrel domain (top) is embedded in the outer membrane, while the C-terminal periplasmic domain (bottom) extends into the periplasmic space. **b** Electrostatic surface potential of the modeled AbOmpA structure, showing the distribution of surface charges across both domains. Red and blue indicate negatively and positively charged regions, respectively. **c** Close-up view of the β-barrel domain, highlighting key extracellular loops labeled (Loop 1–Loop 4). Two views are shown, rotated by 90° to reveal structural details. **d** Side and top views of the periplasmic domain, emphasizing key residues Arg286 and Asp271, which are involved in intramolecular interactions and may play roles in periplasmic signaling. The α-helices are shown in red, β-strands in cyan, and loop regions in white. The structural model was generated using the Schrödinger 2024–3 suite via homology modeling, with *E. coli* OmpA (PDB ID: 1QJP) as the template. Sequence alignment revealed approximately 36.4% identity and 52% similarity between the target and template sequences. The reliability of the predicted structure was further validated by a 100-ns molecular dynamics simulation in explicit solvent, confirming conformational stability with a consistent backbone RMSD (1.5 ~ 2.0 Å) and stable radius of gyration

OmpA was first identified as a major component of the outer membrane in *Escherichia coli* over 4 decades ago [77]. Similarly, AbOmpA is the most abundant protein of the outer membrane in *A. baumannii* [31, 78]. The structure of AbOmpA is predicted to three functional domains: extracellular loops that interact with environmental molecules, a transmembrane domain that facilitates the permeability of small solutes, and an OmpA-like domain that contributes to the stability of the cell wall [79] (Fig. 1). The N-terminal domain of AbOmpA forms an eight-stranded antiparallel β-barrel within the outer membrane, with four extracellular loops extending outward. The C-terminal OmpA-like domain is a globular structure located in the periplasm [80, 81]. While the complete structure of AbOmpA remains unresolved, high-resolution structural data and molecular interactions of the OmpA-like domain with peptidoglycans have been characterized. Specifically, the OmpA-like domain binds non-covalently to diaminopimelate in peptidoglycan through two conserved residues, Asp271 and Arg286, which maintain the integrity of the cell wall [79].

The pore function of OmpA has been well-studied in *E. coli*, where it is known to facilitate the non-specific passage of small molecules. The pore size of *E. coli* OmpA (EcOmpA) is estimated to be 1 nm [82]. EcOmpA exists in two forms: 2–3% of OmpA presents an open channel, while the majority exhibits a closed channel, regulated by a gating mechanism involving the alteration of a salt bridge (from Glu52-Lys82 in the open channel to Glu52-Arg138 in the closed channel) [83]. Additionally, EcOmpA can adopt an alternative conformation, a large 16-stranded β-barrel by adding eight β-strands at the C-terminal domain. This large pore forms when the temperature is elevated [82, 83]. AbOmpA allows the passage of large sugars (up to 500 Da), but it has limited ability to traverse other molecules, implying that the majority of AbOmpA exists as a closed channel. Experimental data demonstrate that AbOmpA is non-specific slow porin for small hydrophilic molecules [78, 84, 85]. The Δ*AbompA* mutants show reduced permeability to cephalosporins compared to the wild-type *A. baumannii* strain. AbOmpA is a selective permeable porin for β-lactamase inhibitor ETX2514, imipenem, and sulbactam [85]. Understanding the structure of AbOmpA is essential for elucidating its pore function and the permeability of molecules as well as its virulence potential.

### Regulation of AbOmpA expression in the outer membrane

The expression of EcOmpA is tightly regulated in response to various environmental and physiological stimuli at the transcriptional, translational, and post-translational levels. However, the regulation of *AbompA* and its expression in the outer membrane have not been well characterized. The expression of AbOmpA is controlled by several regulatory proteins, environmental conditions, and stress responses [86]. Hfq plays a crucial role as a post-transcriptional regulator in bacteria by interacting with small RNAs to influence a wide range of genes [87]. The Hfq-deficient mutants of *A. baumannii* show reduced secretion of AbOmpA, as well as increased interleukin (IL)-8 levels, potentially aiding in bacterial clearance by the host [88]. Additionally, the *A1S_0316* gene encodes a potential transcription factor that regulates *AbompA* expression [89]. When comparing the binding affinities of A1S_0316 and the histone-like nucleoid structuring (H-NS) protein to the *AbompA* promoter region, A1S_0316 exhibits a high affinity, suggesting that it acts as an anti-repressor for the *AbompA* promoter by inhibiting the binding of the H-NS protein.

Environmental factors, such as temperature, osmolarity, ferric ions, and nutrients, influence the expression of AbOmpA. Iron-rich conditions, in particular, promote the expression of *AbompA*, leading to enhanced protein production in *A. baumannii* [90, 91]. Elevation of temperature from 30 to 37 °C, while maintaining a salt concentration of 100 mM, results in a 50% reduction in the expression of total outer membrane proteins. Similarly, a 50% reduction in outer membrane protein expression is observed when the salt concentration increases from 100 to 300 mM NaCl at 30 °C [78]. *A. baumannii* exposed to oxidative stresses, such as ethanol, phenol, cadmium, and hydrogen peroxide, shows high expression of AbOmpA, suggesting that oxidative stress plays a role in regulating AbOmpA expression [92]. Exposure of *A. baumannii* to antibiotics, especially those targeting the cell envelope, such as β-lactams, also increases AbOmpA expression [93]. Colistin and polymyxin B reduces mRNA levels of *AbompA* in *A. baumannii* ATCC 19606^T^ and clinical isolates in a dose-dependent manner [94]. These results suggest that *A. baumannii* regulates AbOmpA expression in the outer membrane at multiple stages in response to environmental and physiological stimuli. However, the mechanism by which membrane-integrated AbOmpA is regulated and secreted as OMV-associated AbOmpA or in free form has not been characterized, and further research is needed.

### Evolutionary conservation of AbOmpA and variability in extracellular loops

AbOmpA shares only 58% homology with the EcOmpA and its homolog in *P. aeruginosa* [95]. However, the *AbompA* gene shows a high degree of nucleotide sequence identity, exceeding 98%, among various *A. baumannii* strains. The size of *AbompA* ranges from 1029 to 1071 nucleotides, while the length of the AbOmpA precursor protein varies between 342 and 356 amino acids [96]. Of the 356 amino acids in AbOmpA, 60 are identified as polymorphic sites across clinical *A. baumannii* isolates. This polymorphism is primarily concentrated in four extracellular loops and terminus of the C-terminal OmpA-like domain. The mutational frequency is highest in extracellular loop 3 (position at 127–151). Amino acid deletions are frequently observed in extracellular loops 1 (position at 36–58), 2 (position at 80–100), and 3, while extracellular loop 4 (position at 176–183) exhibits substitution mutations at three sites. Five distinct AbOmpA variants have been identified within *A. baumannii* populations [96]. The most common variant has a gene length of 1,071 nucleotides, a precursor protein length of 356 amino acids, a mature protein length of 334 amino acids, and specific C-terminal sequences of QEAAAPAAAQ. This variant is found in *A. baumannii* type strain ATCC 19606^T^, ATCC 17978, and 76% of clinical *A. baumannii* isolates. The variations in AbOmpA are closed related to the clonal lineages of *A. baumannii*, with the most frequent AbOmpA variant being associated with global clone 2, the most prevalent epidemic clone worldwide [96]. The high variability in extracellular loops of AbOmpA implies its interaction with a wide range of extracellular molecules. However, this variability may also interfere with the binding of drugs targeting AbOmpA. Mutations at drug-binding sites can diminish its interaction with drugs, potentially rendering AbOmpA-targeting therapies ineffective. Therefore, targeting the highly conserved regions of AbOmpA is essential for developing effective anti-infective agents against *A. baumannii*.

## Comparative analysis of biological functions of the three forms of AbOmpA

*A. baumannii* releases AbOmpA during both in vitro culture and in vivo infection, although AbOmpA is a major outer membrane protein [75, 76]. The majority of AbOmpA is released via OMVs, while a smaller portion remains in the culture supernatant after OMV isolation, representing the free form of AbOmpA [76]. Thus, AbOmpA exists in three forms—outer membrane-integrated, OMV-associated, and free protein—each contributing uniquely to pathogenesis and therapeutic potential. The outer membrane-integrated form plays multiple roles in *A. baumannii* pathogenesis, including host cell interaction [66], biofilm formation [64], surface motility [97], resistance to the host immune response [68, 98], and antimicrobial resistance [81]. These functions make it a promising target for therapeutic intervention [99, 100]. OMV-associated AbOmpA, an abundant protein component of OMVs [75, 76], contributes to host cell death [75], triggers innate immune responses [101, 102], and modulates host immunity [103, 104]. Recombinant AbOmpA (rAbOmpA) like free form of AbOmpA has been shown to bind to host cell the surfaces and be internalized, leading to cytotoxicity and immune activation [66, 105, 106]. Although the free form of AbOmpA may exert effects similar to the OMV-associated form, its overall impact is likely limited due to its lower abundance among secreted proteins [76]. Understanding the distinct biological roles of each form of AbOmpA is crucial for elucidating *A. baumannii* pathogenesis and for developing targeted therapies. While OMV-associated AbOmpA comprises approximately 9% of OMV proteins [76], its pathological role appears distinct from that of the outer membrane-integrated form. OMV-associated AbOmpA likely contributes to pathogenesis through a "remote delivery" mechanism—inducing host cell death and modulating immune responses at distant sites. In contrast, the membrane-integrated form functions locally at the infection site, facilitating host cell interaction, immune evasion, and antimicrobial resistance. Although direct evidence remains limited, these two forms may act synergistically: OMV-associated AbOmpA promotes tissue damage and immune modulation to facilitate bacterial dissemination, while the membrane-integrated form ensures effective colonization and persistence at the infection site.

## Virulence mechanisms of outer membrane-integrated AbOmpA

### Host cell interaction, biofilm formation, and surface motility

Both outer membrane-integrated AbOmpA and OMV-associated AbOmpA can interact with various environmental molecules [107, 108]. During the initial stages of colonization or infection, the extracellular loops of AbOmpA in the outer membrane interact with host cells or abiotic surfaces, such as indwelling catheters and medical devices [66, 99]. rAbOmpA has been shown to specifically bind to the surfaces of different types of human epithelial cells, including lung epithelial cells, laryngeal epithelial cells, and cervical carcinoma cells in vitro [66]. Pretreatment with rAbOmpA significantly reduces the adherence of *A. baumannii* to epithelial cells by over 81%. Although *A. baumannii* is primarily considered an extracellular pathogen, this pathogen can invade non-phagocytic cells via a zipper-like mechanism [66]. The cellular invasion of Δ*AbompA* mutants is dramatically reduced, with a decrease of over 99% compared to wild-type *A. baumannii* ATCC 19606^T^. Additionally, Δ*AbompA* mutants show limited dissemination into the bloodstream in a murine pneumonia model.

Outer membrane-integrated AbOmpA plays a role in biofilm formation of *A. baumannii* [64], although other factors, such as CsuAB/ABCDE-mediated pili and biofilm-associated protein A, have been identified as critical mediators of biofilm formation [109–111]. Disruption of the *AbompA* gene by transposons reduces biofilm formation in *A. baumannii* ATCC 19606^T^ [32]. The ability of Δ*AbompA* mutants of *A. baumannii* ATCC 17978 and clinical isolate 1656–2 to form biofilms is reduced by 4- and 36-fold, respectively, compared to the wild-type strains [64]. Surface-associated motility of Δ*AbompA* mutants is also significantly reduced compared to the wild-type *A. baumannii* ATCC 17978 [97, 112]. Inhibitors of *AbompA* gene expression, such as methoxy-substituted hydroxychalcone, reduce biofilm mass and surface-associated motility in *A. baumannii* [112]. These findings suggest that outer membrane-integrated AbOmpA is a critical effector molecule interacting with host cells and abiotic surfaces. However, further studies are needed to determine which extracellular loops of AbOmpA are responsible for these virulence traits in vitro.

### Resistance to host immune response

The outer membrane-integrated AbOmpA interacts with the fluid-phase complement regulator factor H, inhibiting the activation of the alternative complement pathway and contributing to serum resistance [68]. *A. baumannii* ATCC 19606^T^ maintains viability at 30% normal human serum, whereas the AbOmpA-deficient mutant loses viability at 5% human serum. AbOmpA plays a critical role in the survival of *A. baumannii* in blood, contributing to bacteremia. This protein also influences cellular processes such as autophagy and host immune responses. Autophagy is a conserved cellular process that maintains homeostasis by degrading damaged organelles and pathogens within autophagosomes. *A. baumannii* has developed sophisticated strategies to either exploit or disrupt this process to ensure its survival and proliferation. AbOmpA activates signaling pathways such as mammalian target of rapamycin (mTOR) and mitogen-activated protein kinases (MAPK)/JNK to induce autophagy in host cells but disrupts the fusion of autophagosome with lysosomes, preventing bacterial clearance [98]. This disruption leads to the accumulation of autophagosomes, creating a protective intracellular environment that enhances bacterial survival. Previous study have shown that AbOmpA increases key autophagy markers such as LC3-II while inhibiting the degradation of p62, thus inducing incomplete autophagy [98]. In addition, AbOmpA promotes dendritic cell (DC) maturation, as evidenced by increased expression of major histocompatibility complex (MHC)-II, CD80, and CD86 [113]. It also stimulates the secretion of pro-inflammatory cytokines, such as IL-1β, tumor necrosis factor (TNF)-α, and IL-18, and induces apoptosis. The phosphoinositide 3-kinase (PI3K)/mTOR pathway is a key mediator of these effects, with overexpression of PI3K suppressing the expression of autophagy marker and reducing inflammation and apoptosis [70]. The incomplete autophagy induced by AbOmpA not only facilitates the intracellular survival of *A. baumannii* but also exacerbates tissue damage and disease progression by triggering the release of pro-inflammatory cytokines like IL-1β. The ability of AbOmpA to modulate autophagy is closely associated with the pathogenicity of *A. baumannii*. By inducing incomplete autophagy, *A. baumannii* evades immune surveillance, establishing a persistent intracellular infection and promoting antimicrobial resistance. Furthermore, the induction of apoptosis and inflammation accelerates tissue damage and bacterial dissemination, highlighting the critical role of AbOmpA in the pathogenesis of *A. baumannii* [114]. Targeting the effects of AbOmpA on autophagy represents a promising therapeutic strategy. Autophagy modulators, such as rapamycin, may restore disrupted autophagic flux caused by AbOmpA and enhance the clearance of intracellular bacteria. Additionally, designing small molecule inhibitors or antibodies to block AbOmpA activity could reduce bacterial survival and decrease infection severity. Combining these approaches with existing antimicrobial agents may yield synergistic effects, providing a comprehensive solution to combat *A. baumannii* infections. Further studies on the molecular interactions between AbOmpA and autophagic pathways could uncover new therapeutic targets and deepen our understanding of host–pathogen interactions.

## Contribution of outer membrane-integrated AbOmpA to antimicrobial resistance

The deletion or disruption of *AbompA* gene affects the antimicrobial susceptibility of *A. baumannii* [81]. Tn*26*-inserted *AbompA* mutants of *A. baumannii* AB5075 show a fourfold decrease in minimum inhibitory concentrations (MICs) against ampicillin/sulbactam, piperacillin, cefepime, aminoglycosides, and levofloxacin, while demonstrating a fourfold increase in MIC against tetracycline [100]. The Δ*AbompA* mutants of *A. baumannii* ATCC 17978 and clinical strain 1656–2 exhibit increased susceptibility to trimethoprim (≥ fourfold) compared to the wild-type strains [81]. Complementation of *AbompA* and its promoter region in the Δ*AnompA* (*ompA* of *A. nosocomialis*) mutant of *A. nosocomialis* ATCC 17903 results in a > fourfold increase in MIC against trimethoprim compared to the wild-type strain [115]. The OmpA-like domain-deleted mutants of *A. baumannii* ATCC 17978 show ≥ twofold reduction in MICs against aztreonam, colistin, gentamicin, imipenem, and trimethoprim compared to the wild-type strain [81]. However, no difference in the MICs is observed between the wild-type and OmpA-like domain-deleted mutant strains for gentamicin, nalidixic acid, and tetracycline when efflux pump inhibitor phenylalanine-arginine β-naphthylamide is present. Wu et al. [116] proposes a potential interaction between AbOmpA and AdeK, a member of the AdeIJK RND efflux pump system. AbOmpA contributes to intrinsic resistance to clinically important antimicrobial agents through its OmpA-like domain [117]: however, its interaction with RND efflux pumps requires further investigation.

## AbOmpA and OMVs

### Packing of AbOmpA in *A. baumannii* OMVs

All living cells, both eukaryotic and prokaryotic cells, release extracellular vesicles (EVs) into the extracellular environment [118]. Both Gram-positive and Gram-negative bacteria produce EVs during their growth. Bacterial EVs are spherical structures that vary in size, ranging from 20 to 400 nm in diameter [119, 120]. These EVs play crucial roles in bacterial survival, cell–cell communication, and pathogenesis by delivering effector molecules to host cells [121–123]. Gram-negative bacteria release EVs from their outer membrane, referred to as OMVs [124]. In addition, outer-inner membrane vesicles and tube-shaped membranous structures have been proposed by the different routes of MV formation [119]. *A. baumannii* produces and releases OMVs during both in vitro culture and in vivo infection [67, 75]. The average size of OMVs from *A. baumannii* ATCC 17978 ranges from 183.9 to 193.7 nm [125]. AbOmpA is one of the most abundant proteins in the supernatants of *A. baumannii* during in vitro culture [76]. The majority of AbOmpA released from *A. baumannii* is associated with OMVs [75]. Quantitative proteomic analysis demonstrates that AbOmpA is one of the abundant proteins present in *A. baumannii* OMVs. The full-length AbOmpA has been identified in *A. baumannii* OMVs. AbOmpA is exclusively located in the vesicular membrane, not in the lumen of the OMVs [75]. Incorporating AbOmpA into OMVs preserves its native β-barrel conformation, offering several advantages, including protection from degradation, improved delivery and targeting, coordinated secretion with other effectors, and exposure to a unique microenvironment that may enhance its functional activity [75, 108]. Other virulence factors, in addition to AbOmpA, and biologically active enzymes such as β-lactamases have been found in *A. baumannii* OMVs [126]. OMVs released from *A. baumannii* interact with cholesterol-rich membrane microdomain (lipid rafts) in the cytoplasmic membrane of host cells, facilitating the delivery of AbOmpA to the cytoplasm [75]. When epithelial cells are exposed to *A. baumannii* OMVs in vitro, full-length AbOmpA is detected in the cytoplasm of host cells within 30 min and remains there for over 12 h. *A. baumannii* OMVs serve as an efficient vehicle for delivering AbOmpA and other bacterial molecules to host cells, where they interact with cellular components and contribute to various pathological processes.

### Regulation of OMV production

OMV production is apparently increased under harsh environmental conditions, such as nutrient restriction, chemical exposure, antibiotic treatment, extreme pH, or host infection, although the biogenesis of bacterial EVs has not been fully elucidated [127, 128]. *A. baumannii* markedly increases OMV production under stress conditions, including exposure to hydrogen peroxide, D-cycloserine, and antimicrobial agents like polymyxin B, colistin, and imipenem [129–131]. When *A. baumannii* is exposed to subinhibitory concentrations of imipenem, the proteome components of OMVs are modified. More AbOmpA (1.57-fold increase) is packaged in OMVs from *A. baumannii* exposed to imipenem compared to those cultured without antibiotics. Moreover, two-component regulatory systems, such as PmrAB and BfmRS, also govern OMV production in *A. baumannii* [62, 125]. The Δ*pmrA* and Δ*pmrB* mutants produce less number of OMVs compared to wild-type *A. baumannii* ATCC 17978 [62]. Conversely, the Δ*bfmS* mutant produces more OMV particles than the wild-type *A. baumannii* ATCC 17978 [125]. The zinc uptake regulator-regulated lipoprotein A (ZrlA) plays a role in overcoming antimicrobial expose and pathogenesis in *A. baumannii* [132]. The Δ*zrlA* mutants produce 9.7-fold more OMV particles than the wild-type strain, although the size and protein profile of OMVs remain similar between the wild-type and mutant strains. However, OMVs from the Δ*zrlA* mutant are more cytotoxic towards epithelial cells than those from the wild-type strain. Since AbOmpA is a cytotoxic factor [31], its abundance in OMVs contributes to increased host cell cytotoxicity. ZrlA exhibits peptidase activity, suggesting that Δ*zrlA* mutants inhibit peptidoglycan remodeling and reduce crosslinking between peptidoglycans and the outer membrane, leading to hyperproduction of OMVs [48]. Additionally, AbOmpA in the outer membrane modulates OMV biogenesis through its interaction with peptidoglycan. The Δ*AbompA* mutants produce 13-fold and sevenfold more proteins and LOS in OMVs, respectively, than the wild-type *A. baumannii* ATCC 19606^T^ [67]. Various stressful conditions and regulatory systems for environmental adaptation influence the virulence of *A. baumannii* by regulating OMV production and the packing of AbOmpA in OMVs.

## Pathological roles of OMV-associated AbOmpA

### Host cell death

**Fig. 2:**
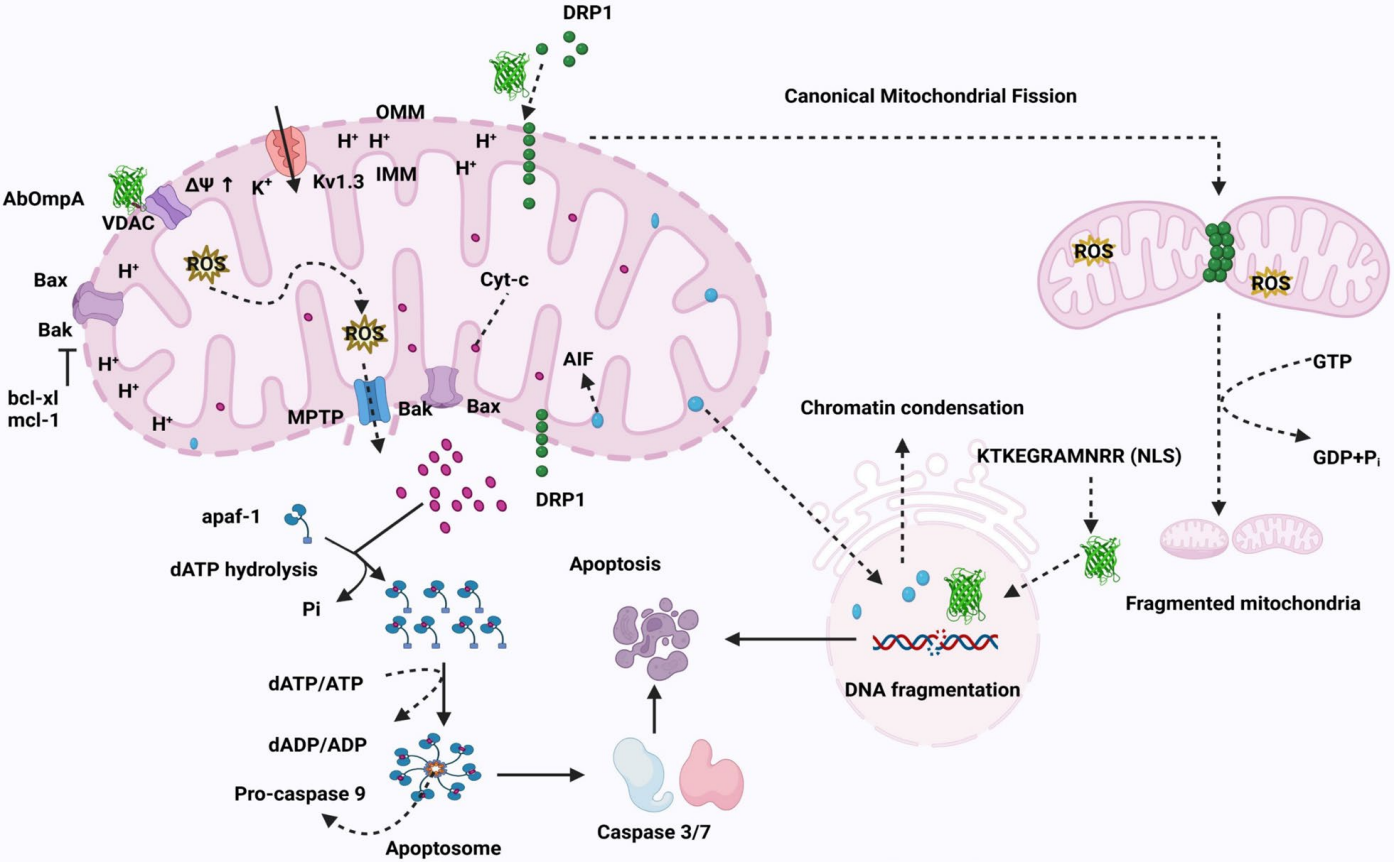
Schematic representation of host cell death induced by OMV-associated AbOmpA. AbOmpA binds to the VDAC on the mitochondrial outer membrane. This leads to an increased mitochondrial transmembrane potential (ΔΨ), ROS production, and inner membrane hyperpolarization. These events trigger the opening of the mitochondrial permeability transition pore (MPTP), resulting in mitochondrial swelling, loss of membrane integrity, and cytochrome *C* release, which initiates apoptotic cascade. In parallel, AbOmpA activates DRP1, promoting its accumulation on mitochondria, further enhancing ROS production and mitochondrial fragmentation. Released cytochrome *C* activates apoptotic protease activating factor 1 (Apaf1), forming the apoptosome complex and subsequently activating caspase-3 and -7 to execute apoptosis. Upon mitochondrial outer membrane rupture, apoptosis inducing factor (AIF) is translocated to the nucleus, contributing to apoptosis. Additionally, AbOmpA can enter the nucleus via its nuclear localization signal (NLS), where it degrades DNA through its DNase I activity. Biorender software (Biorender.com) was used to create figure

AbOmpA induces host cell death through both early-onset apoptosis and delayed-onset necrosis [105]. When epithelial cells are exposed to rAbOmpA, both the full-length and subfragments of rAbOmpA are detected in the cytoplasm within 4–8 h, after which subfragments of rAbOmpA move to the nuclei [133]. The cytoplasmic rAbOmpA binds to the voltage-dependent anion channel (VDAC) in the outer membrane of mitochondria (unpublished data). As VDAC is a component of the outer membrane permeability transition pore, its interaction with AbOmpA triggers mitochondrial permeability transition. This event leads to an increase in mitochondrial transmembrane potential, which subsequently induces the generation of reactive oxygen species (ROS), followed by mitochondria swelling and rupture of the outer membrane [31, 105] (Fig. 2). These sequential events trigger the release of pro-apoptotic molecules, such as cytochrome *C* and apoptosis-inducing factor, from the intermembrane space of mitochondria into the cytoplasm, activating caspase-dependent apoptosis and macromolecular DNA digestion in host cells of host cells, respectively, during the early stages [31, 105, 133]. ROS generated from the mitochondria also contributes to the delayed-onset necrosis of host cells [105]. Furthermore, two subfragments of rAbOmpA with 27 and 30 kDa are detected in the nuclei of host cells [133]. The nuclear targeting of AbOmpA is facilitated by nuclear localization signals (KTKEGRAMNRR) located in the C-terminal OmpA-like domain, which mediates the transport of AbOmpA to the nucleus of host cells through the nuclear pore complex [69]. This nuclear localization induces DNA fragmentation of host cells through DNase I-like activity [133]. While nuclear localization of AbOmpA is predominantly observed in epithelial cells, it is rarely seen in DCs [105]. Although AbOmpA can target both mitochondria and nuclei of host cells, mitochondrial targeting is the primary pathway for inducing cell death.

OMVs from *A. baumannii* ATCC 17978 induce host cell death, whereas OMVs from the Δ*AbompA* mutants do not [75], indicating that AbOmpA in the OMVs is a critical cytotoxic factor responsible for host cell death. Like *A. baumannii* OMVs, OMVs derived from *A. nosocomialis* ATCC 17903 induce host cell cytotoxicity, while OMVs from Δ*AnompA* mutants do not, indicating that AnOmpA is a potential cytotoxic factor in OMVs [134]. Tiku et al. [135] recently reported that AbOmpA in OMVs derived from *A. baumannii* strains induces mitochondrial fragmentation through the activation of GTPase dynamin‑related protein 1 (DRP1). The activation of DRP1 by OMV-associated AbOmpA leads to its accumulation in mitochondria, where it oligomerizes and assembles into spiral structures on the outer mitochondrial membrane in a GTP-dependent process and induces canonical mitochondrial fragmentation in an AbOmpA-dependent manner (Fig. 2). Subsequently, ROS originating from mitochondria induces host cell death. In a murine *A. baumannii* infection model, AbOmpA induces mitochondrial fragmentation in alveolar macrophages, while infection with the Δ*AbompA* mutants does not result in mitochondrial fragmentation in these cells [135].

AbOmpA induces host cell death in a dose-dependent manner. A concentration of 6 µg/ml (protein concentrations) of purified AbOmpA from *A. baumannii* ATCC 19606^ T^ is sufficient to induce epithelial HEp-2 cell death [66]. However, lethal concentrations of rAbOmpA vary between host cell types: ≥ 15 µg/ml in epithelial HEp-2 cells, ≥ 6 µg/ml in U937 macrophages, and ≥ 3 µg/ml in murine bone marrow-derived DCs [71, 75, 106]. Additionally, ≥ 50 µg/ml of OMVs purified from *A. baumannii* ATCC 19606^T^ and clinical isolate DU202 induces cell death in HEp-2 and A549 cells, respectively [75, 129]. Skerniškytė et al. [102] reported that only 4 µg/ml of OMVs from the clinical *A. baumannii* Ab_169_ strain induces cytotoxicity in murine J774 macrophages and human lung epithelial A549 cells. *A. baumannii* OMVs are more cytotoxic to murine macrophages than A549 cells. Furthermore, OMVs from the Δ*AbompA* mutants exhibit reduced cytotoxicity compared to those from the wild-type strain. Immune cells exhibit a lower threshold for cell death induced by AbOmpA and AbOmpA-containing OMVs compared to epithelial cells. OMVs purified from *A. baumannii* DU202 cultured with sublethal concentrations of imipenem or tetracycline are more cytotoxic than OMVs from *A. baumannii* cultured without antibiotics [129]. The OMVs from the Δ*bfmS* mutant are more cytotoxic to lung epithelial A549 cells than those from the wild-type strain, due to the higher concentrations of AbOmpA in the OMVs from the Δ*bfmS* mutant [125]. The concentrations of AbOmpA in the OMVs vary depending on bacterial culture conditions or two-component regulatory systems.

Micelles composed of rAbOmpA_1-356_ and rAbOmpA_22-170_, which mimic the AbOmpA containing OMVs, induce cell death in U937 macrophages at concentrations of ≥ 5 µg/ml, whereas micelles composed of rAbOmpA_221-339_ do not induce host cell death at 100 µg/ml [75]. The N-terminal domain of AbOmpA is responsible for inducing host cell death, although the specific binding sites of AbOmpA to the voltage-dependent anion channel in the outer membrane of mitochondria and cellular DNA have not yet been identified. It is evident that OMV-associated AbOmpA induces host cell death both in vitro and in vivo; however, the impact of this cell death during *A. baumannii* infection remains to be elucidated.

### Induction of innate immune response

**Fig. 3:**
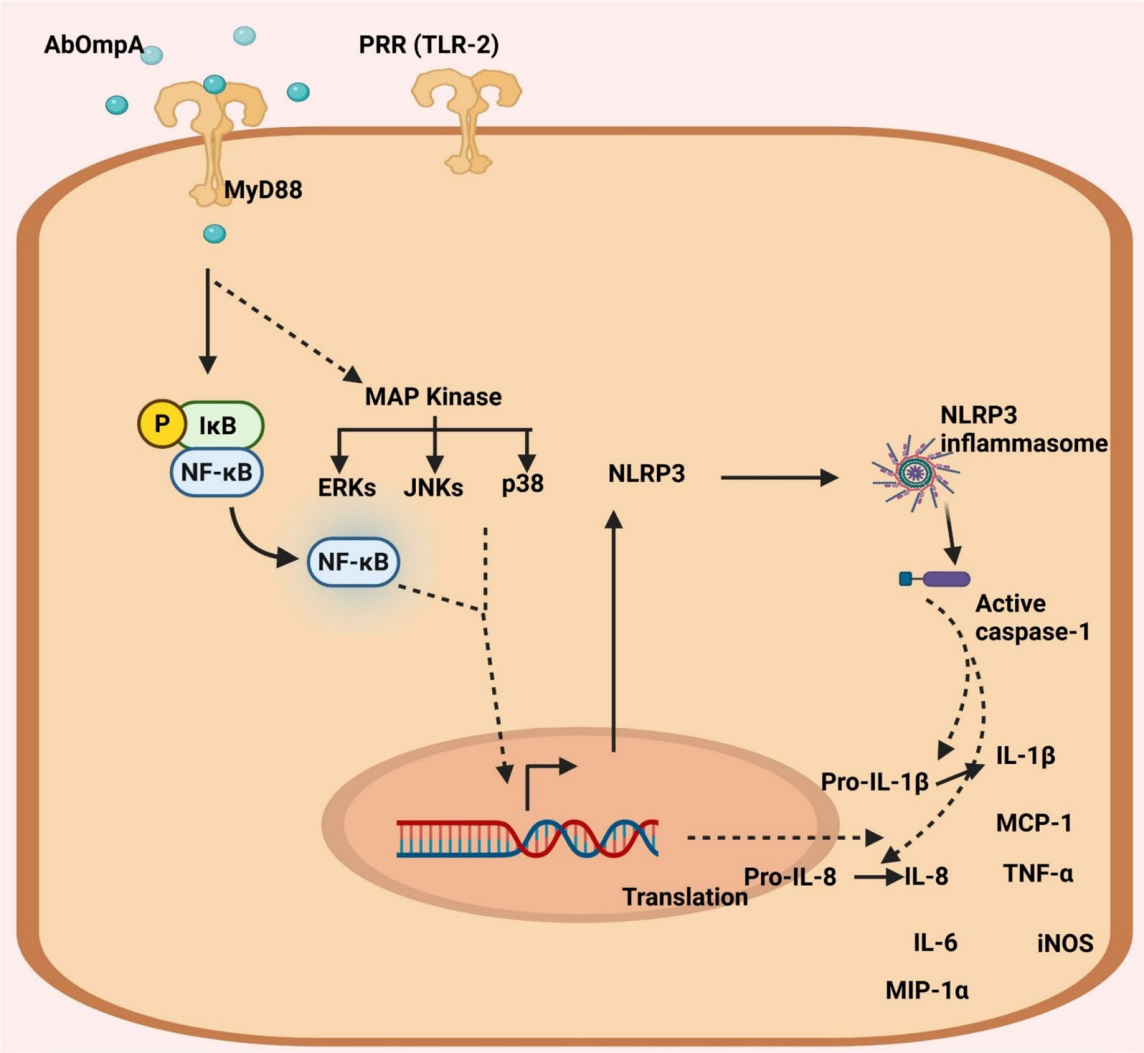
Innate immune response induced by *A. baumannii* OMVs and OMV-associated AbOmpA. *A. baumannii* OMVs elicit a pro-inflammatory response, primarily mediated by AbOmpA. AbOmpA interacts with the cell surface receptor TLR2, leading to recruitment of the adaptor protein MyD88 and activation of the NF-κB and MAPK signaling pathways, including ERKs, JNKs, and p38. This signaling cascade promotes the transcription of genes encoding pro-inflammatory cytokines and chemokines, such as IL-1β, IL-6, IL-8, MCP-1, TNF-α, and MIP-1α. Additionally, activation of the NLRP3 inflammasome leads to the cleavage of procaspase-1 into active caspase-1, which subsequently processes pro-IL-1β into its active form, amplifying the inflammatory response. Biorender software (Biorender.com) was used to create figure

Microarray analysis revealed that sublethal concentrations of rAbOmpA differentially regulated a total of 242 genes in epithelial HEp-2 cells [71]. Among the differentially regulated genes, genes associated with signal transduction pathways are the most prevalent, followed by immune and inflammatory response genes. rAbOmpA upregulates pro-inflammatory cytokine (IL-1β, IL-6, and TNF-α), chemokine (IL-8), and inducible nitric oxide synthase genes. Additionally, rAbOmpA increases the surface expression of Toll-like receptor 2 (TLR2), making host cells more responsive to its ligand. rAbOmpA activates MAPKs, including ERKs, JNKs, and p38, and NF-κB [71].The NOD-like receptor protein 3 (NLRP3) inflammasome plays a crucial role in stimulating and regulating the inflammatory response by activating caspase-1 and promoting the secretion of IL-1β [136]. *A. baumannii* activates the NLRP3 inflammasome, enhancing inflammation and lung damage during pulmonary infection [137]. AbOmpA inhibits the degradation of caspase-1 and promotes the assembly of the NLRP3 inflammasome through the TLR2-NF-κB pathway, leading to the maturation of IL-1β and other pro-inflammatory molecules [138] (Fig. 3). Overall, AbOmpA is a potent inducer of the innate immune response both in vitro and in vivo.

Bacterial EVs, including *A. baumannii* OMVs, contain a variety of pathogen-associated molecular patterns (PAMPs) that induce innate immune responses, contributing to both bacterial clearance and immunopathology during bacterial infection [101, 139]. *A. baumannii* OMVs carry LOS, ≥ 100 different proteins, and nucleic acids. All components of OMVs, including AbOmpA, can act as PAMPs, which are recognized by various pattern recognition receptors located in the cytoplasmic membrane or cytosol of host cells [139]. Sublethal concentrations of *A. baumannii* OMVs upregulate the expression of pro-inflammatory cytokine genes (IL-1β and IL-6), and chemokine genes (IL-8, macrophage inflammatory protein-1α, and monocyte chemoattractant protein-1) in epithelial cells [101]. Furthermore, *A. baumannii* OMVs induce inflammatory responses, including significant leukocyte infiltration and the expression of pro-inflammatory cytokine genes in the skin and lungs of mice in vivo. Disintegration of OMVs by treating ethylene-diamine-tetraacetic acid results in decreased expression of pro-inflammatory cytokine genes compared to intact OMVs. The expression levels of pro-inflammatory cytokine genes are similar between epithelial cells treated with proteinase K-treated OMVs (which partially digest vesicular membrane proteins) and untreated control cells, suggesting that the surface-exposed proteins in the OMVs are directly responsible for inducing the pro-inflammatory response. OMVs from *A. baumannii* ATCC 19606^T^ and its isogenic Δ*AbompA* mutant exhibit comparable expression levels of pro-inflammatory cytokine genes in HEp-2 cells [134]. However, another study by Skerniškytė et al. [102] reported that AbOmpA-deficient OMVs from the clinical isolate Ab_169_ can trigger a pro-inflammatory response in murine macrophages, although the expression levels of TNF-α, IL-6, IL-1β, and NLRP3 genes in macrophages treated with AbOmpA-deficient OMVs are lower than those in cells treated with AbOmpA-carrying OMVs from the wild-type strain. OMV-associated AbOmpA plays a crucial role in inducing inflammatory responses. As *A. baumannii* adapts to its environment, it alters the production and composition of OMVs, which can trigger different innate immune responses [140].

### Immune modulation

**Fig. 4:**
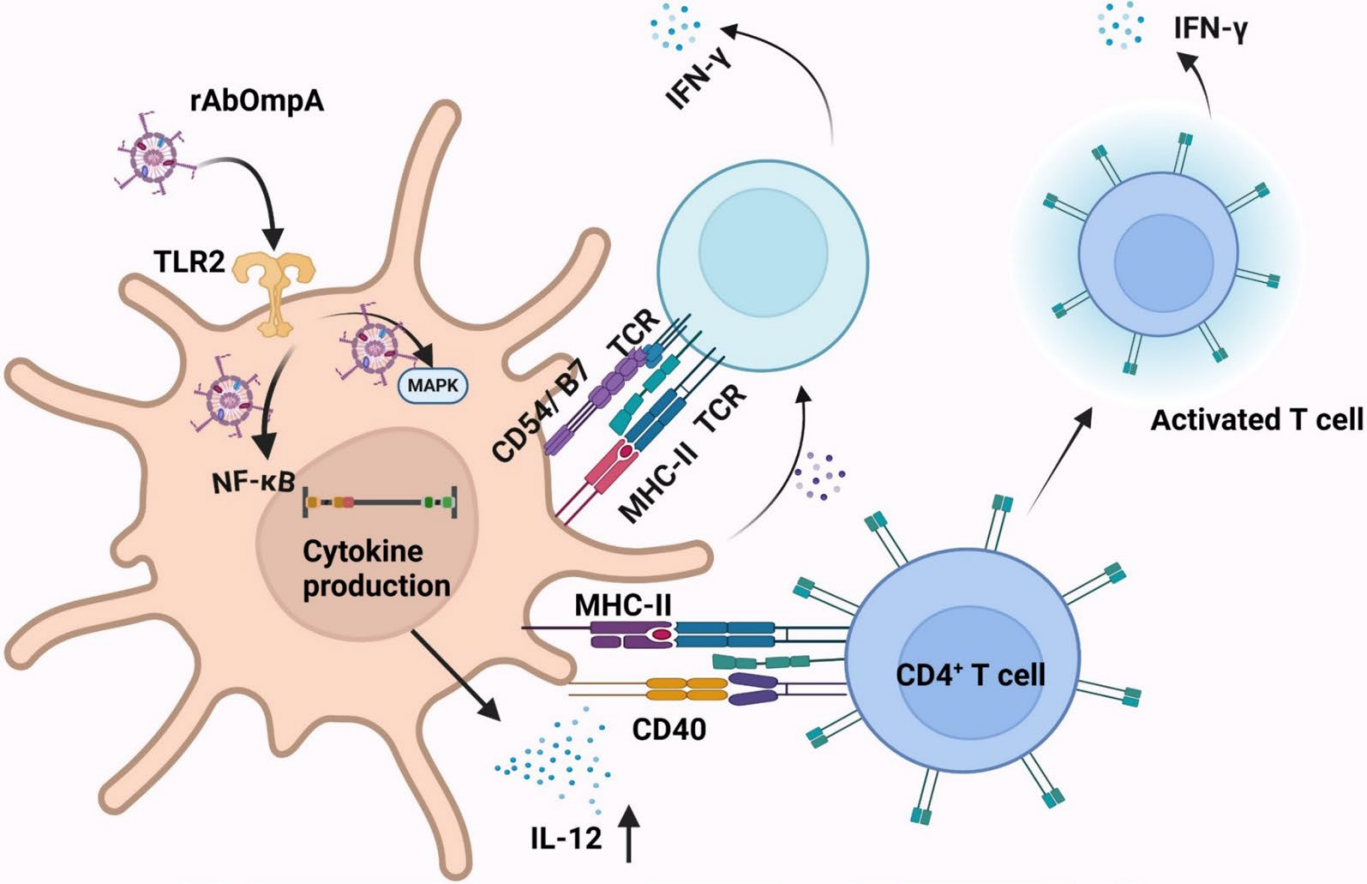
Immune modulation by *A. baumannii* OMVs and OMV-associated AbOmpA. *A. baumannii* OMVs, and particularly OMV-associated AbOmpA, modulate the adaptive immune response by interacting with antigen-presenting cells, such as DCs. AbOmpA binds to pattern recognition receptors (PRRs) on antigen-presenting cells (APCs), triggering signaling pathways that upregulate antigen-presenting molecules, including MHC class II, as well as co-stimulatory molecules such as CD40, CD54, and B7. These activated APCs present OMV-derived antigens to CD4^+^ T cells, promoting TH1 cell activation and proliferation. In turn, activated TH cells stimulate B cells, driving their differentiation into plasma cells that secrete OMV-specific antibodies. During this process, APCs also release pro-inflammatory cytokines, including IL-12 and IFN-γ, further enhancing the immune response. Biorender software (Biorender.com) was used to create figure

DCs are the most potent antigen-presenting cells, linking innate and adaptive immunity [141]. Immature DCs can capture and process *A. baumannii*, its byproduct, and OMVs in tissues that interface with the external environment, such as the lungs and skin. While high concentrations of rAbOmpA (≥ 3 µg/ml of proteins) induce DC death, low concentrations (100 and 200 ng/ml of proteins) activate DCs through the TLR2-mediated signaling pathway, as evidenced by the reduced IL-12 production in DCs pretreated with a TLR2 blocking peptide [105, 106] (Fig. 4). The binding of rAbOmpA to TLR2 activates MAPKs and NF-κB, leading to the maturation and activation of DCs [115]. rAbOmpA significantly increases the expression of surface markers, including CD40, CD54, B7 family, and MHC class I and II, in murine bone marrow-derived DCs in vitro and splenic DCs in vivo. The expression levels of these surface markers are higher in rAbOmpA-treated DCs than those in LPS-treated DCs. Additionally, DCs activated by rAbOmpA secrete IL-12, but not IL-10, promoting the differentiation of naïve CD4^+^ T cells to Th1 CD4^+^ T cells [106, 113]. Mature DCs activated by rAbOmpA stimulate the proliferation and interaction with naïve T cells. T cells interacting with rAbOmpA-activated DCs secret large amounts of IFN-γ, which drives a Th1 immune response [106]. The N-terminal region of AbOmpA is predicted to be more immunogenic than the C-terminal OmpA-like domain [142]. Peptides located at positions 24–50 of AbOmpA, specifically VTVTPLLLGYTFQDSQHNNGGKDGNLT, are predicted to contain B and T cell epitopes that elicit a strong humoral immune response similar to that of AbOmpA [142]. These findings indicate that AbOmpA plays a significant role in shaping the adaptive immune response during *A. baumannii* infection.

*A. baumannii* OMVs are enriched with protein PAMPs, including AbOmpA [75, 76]. It is evident that AbOmpA recognized by DCs skews the adaptive immune response towards Th1 immunity; however, the presence of B cell epitopes in AbOmpA and other OMV proteins may influence the balance between Th1 and Th2 immune responses against *A. baumannii* OMVs [106]. Several studies have demonstrated that purified *A. baumannii* OMVs induce a protective immune response against *A. baumannii* infection by producing high levels of OMV-specific IgG antibodies [143]. Furthermore, immunization of mice with *A. baumannii* OMVs produce high level of AbOmpA-specific IgG [103, 104]. Overall, OMV-associated AbOmpA skews the adaptive immune response towards Th1 immunity by DCs and it also induce Th2 immunity by producing specific antibodies. Whether OMVs shed from *A. baumannii* during bacterial infection elicit protective immunity or contribute to immunopathology remains to be elucidated. Although OMV-associated AbOmpA plays a role in induction of innate and adaptive immune responses, its effects on immune tolerance and regulatory T cell responses are not yet well understood. Further research is needed to explore AbOmpA’s potential involvement in activating tolerogenic pathways and regulating immune responses.

## New anti-infective agents targeting AbOmpA

### Small molecules, small synthetic peptides, and antimicrobial peptides

An anti-virulence strategy is an alternative approach that focuses on blocking bacterial virulence rather than destroying essential bacterial functions [144]. Although the development of specific anti-infective agents targeting AbOmpA is still in the early stages, several therapeutic compounds, such as small molecules, peptide-based inhibitors, and monoclonal antibodies, have been investigated (Table 1). Achieving no or low expression of the *AbompA* gene is a promising strategy for developing anti-infective agents to combat *A. baumannii*. Small molecules that bind to the *AbompA* promoter can inhibit its transcription and the production of AbOmpA, thereby reducing the virulence of *A. baumannii* mediated by both outer membrane-integrated AbOmpA and OMV-associated AbOmpA. Na et al. [145] developed high-throughput screening platforms using the reporter strain of *A. baumannii* ATCC 17978 carrying the *AbompA* promoter and *nptI* fusion plasmids. The expression of *nptI* gene conferring resistance to kanamycin is controlled by the activity of the *AbompA* promoter. Small molecules that binds to the promoter inhibit the growth of the reporter strains in the presence of kanamycin. Using this platform, three small molecules (223,604, 195,925, and 62,520) were identified as partial inhibitors of *AbompA* expression and AbOmpA production in the outer membrane of *A. baumannii* ATCC17978. These small molecules reduced biofilm formation of *A. baumannii* ATCC 17978. Further study revealed that small molecule 62,520 significantly enhanced the survival of both immunocompetent and neutropenic mice infected with *A. baumannii* ATCC 17978 and clinical CRAB isolate [146]. A plant alkaloid, tryptanthrin, and methoxy-substituted hydroxychalcone have been shown to significantly inhibit biofilm formation of *A. baumannii* by down-regulation of biofilm-related genes, including *AbompA* [112, 147]. Limonene is an essential oil found in plants. Limonene-loaded alginate/collagen nanoparticles decrease the transcription of *AbompA* and *bap* genes, leading to potent antimicrobial and anti-biofilm activities [148]. Inhibitors that suppress the transcription of *AbompA* can block the virulence of *A. baumannii* by both outer membrane-integrated AbOmpA and OMV-associated AbOmpA.

**Table 1:** Anti-infective agents targeting AbOmpA

Agents	Structure	Molecular formula/sequences	Antibacterial mechanism	Activity	Toxicity
Small molecules					
223604		C_13_H_9_BrN_2_	Inhibition of *AbompA* expression	35% inhibition of *AbompA* expression and 70% inhibition of biofilm mass in *A. baumannii* ATCC 17978 at 1 μM [145]	No toxicity in U937 cells at ≤ 10 μM [145]
195925		C_13_H_12_ClFN_2_O_3_	Inhibition of *AbompA* expression	80% inhibition of *AbompA* expression and 70% inhibition of biofilm mass in *A. baumannii* ATCC 17978 at 1 μM [145]	No toxicity in U937 cells at ≤ 10 μM [145]
62520		C_13_H_11_FN_2_O_4_	Inhibition of *AbompA* expression at sub-MIC and bacteriostatic activity	Inhibition of *AbompA* expression and biofilm mass in *A. baumannii* ATCC 17978 at ≥ 0.35 µg/ml in a dose-dependent manner. Increasing the survival rates of mice at 2.8 mg/kg [146]	No toxicity in U937 cells at ≤ 10 μM [145]
Alkaloid					
Tryptanthrin		C_15_H_8_N_2_O_2_	Inhibition of *AbompA* and other biofilm-associated gene expression	Inhibition of *AbompA* expression and biofilm mass in *A. baumannii* ATCC 17978 at ≥ 10 µg/ml in a dose-dependent manner. [147]	< 5% red blood cell hemolysis at 100 µg/ml [147]
Organic compound					
2ʹ,6ʹ-dihydroxy 4ʹ-methoxy dihydrochalcone		C_16_H_16_O_4_	Inhibition of *AbompA* and other biofilm-associated gene expression	Inhibit biofilm in *A. baumannii* ATCC 19606^ T^ at ≥ 35 µg/ml [112]	Not tested
Cyclic monoterpene					
Limonene		C_10_H_16_	Inhibition of *AbompA* and *bap* expression	Inhibition of *AbompA* expression and biofilm mass in *A. baumannii* strains [148]	Cytotoxicity in HEK293 cells at 12.5 µg/ml [148]
Polypeptides					
P92		QMGFMTSPKHSV	Binding to AbOmpA	Reduction of *A. baumannii* virulence mediated by outer membrane-integrated AbOmpA at ≥ 0.1 µM in a dose-dependent manner [149]	Not tested
AOA-2		C_44_H_58_N_14_O_6_	Binding to AbOmpA	Reduction of *A. baumannii* virulence mediated by outer membrane-integrated AbOmpA at ≥ 0.25 mg/ml. Increasing the survival of mice at 10 mg/kg [150]	No mice toxicity at ≤ 40 mg/kg [150]
Antimicrobial peptides					
BMAP-28		GGLRSLGRKILRAWKKYGPIIVPIIRI	Binding to AbOmpA	MICs of 5–10 µg/ml in pandrug-resistant *A. baumannii* isolates [153]	Not tested
LL-37		LLGDFFRKSKEKIGKEFKRIVQRIKDFLRNLVPRTES	Binding to AbOmpA_74-84_	Bactericidal activity in *A. baumannii* ATCC 17978 at ≥ 2.5 µg/ml in a dose-dependent manner [154]	Not tested
HD5d5		ARARCRRGRAARRRRLRGVCRIRGRLRRLAAR	Binding to AbOmpA	MIC of 40 µg/ml in MDR *A. baumannii.* Increasing the survival rate of mice at 100 µg/ml mg/kg [155]	No cytotoxicity in erythrocytes and HaCaT cells at 100 µg/ml [155]

Neutralizing AbOmpA by small polypeptides is another strategy to develop anti-infective agents against *A. baumannii*. A small peptide, P92 (QMGFMTSPKHSV), has been identified through phage display screening as a strong binder to AbOmpA [149]. P92 effectively reduces *A. baumannii* virulence, mediated by outer membrane-integrated AbOmpA, including bacterial adherence and invasion, and biofilm formation. A significant positive relationship was found between the antibacterial activity of P92 and the expression levels of AbOmpA. Additionally, a synthetic cyclic hexapeptide, AOA-2, and its optimized derivatives specifically bind to AbOmpA, reducing adherence to host cells and biofilm formation [150]. These cyclic peptides also show synergistic activity with colistin against *A. baumannii* [151, 152]. In vivo animal studies, AOA-2 significantly reduces bacterial load in the spleen and lungs and greatly decreases mortality in mice infected with *A. baumannii*. These synthetic peptides interact with negatively charged membranes electrostatically, leading to a permeabilization of the cell membrane and leakage of the cellular contents.

Several antimicrobial peptides (AMPs), including BMAP-28, LL-37, and HD5d5, have been found to exhibit antimicrobial activity against *A. baumannii* [153–155]. A bovine myeloid antimicrobial peptide, BMAP-28, exhibits strong and rapid bactericidal activity against pandrug-resistant *A. baumannii* [153]. The antibacterial activity of BMAP-28 is mediated by a specific interaction with AbOmpA. Human cathelicidin-derived LL-37 specifically interacts with AbOmpA_74-84_ peptides, leading to reduced bacterial surface motility and adherence to host cells [154]. A derivative of human defensin-5, HD5d5, exhibits antibacterial activity against *A. baumannii* through binding to AbOmpA both in vitro and in vivo [155]. Moreover, HD5d5 enters the cytoplasm of *A. baumannii*, where it interacts with cytoplasmic molecules and decreases the activities of superoxide dismutase and catalase, causing the accumulation of ROS. Phytochemicals and chiral phthalimides that specifically bind to AbOmpA have demonstrated antimicrobial activity against *A. baumannii *in vitro [156, 157]. While small synthetic polypeptides and AMPs can bind to both outer membrane-integrated AbOmpA and OMV-associated AbOmpA, their neutralizing activity is primarily attributed to interaction with outer membrane-integrated AbOmpA.

### Perspective into anti-infective agents targeting AbOmpA

Therapeutic failure in patients infected with *A. baumannii* is primarily attributed to antimicrobial resistance, but persister cells that tolerate lethal concentrations of antimicrobial agents also contribute to treatment failure [158]. Although the formation of persisters in *A. baumannii* has not been fully characterized, AbOmpA is involved in persister formation, as the *AbompA* gene is upregulated by ≥ 5.5-fold in *A. baumannii* persisters induced by meropenem exposure [158]. Thus, AbOmpA represents a promising therapeutic target not only for actively replicating cells but also for *A. baumannii* persisters.

The selection of specific target domains or sites is crucial for the development of AbOmpA-neutralizing agents. The extracellular loops of the outer membrane-integrated AbOmpA are responsible for host cell adherence and invasion, dissemination of bacteria into blood, serum resistance, biofilm formation, surface motility, and mortality of mice infected with *A. baumannii* (unpublished data), although the pathogenic roles of each extracellular loop have not been characterized. Additionally, surface-exposed membrane proteins, including AbOmpA, present in *A. baumannii* OMVs are directly responsible for triggering pro-inflammatory cytokine responses in epithelial cells [101]. Since all four extracellular loops of AbOmpA are exposed on the surface of bacteria or OMVs, they are easily accessible for targeting by small molecules or peptides. However, polymorphisms in AbOmpA, particularly in extracellular loop 3, are frequently observed in clinical *A. baumannii* strains [96]. Consequently, it is crucial to design small molecules or peptides that selectively bind to the conserved regions of these extracellular loops to effectively neutralize both outer membrane-integrated AbOmpA and OMV-associated AbOmpA during bacterial infection. The OmpA-like domain of AbOmpA presents another promising target for combating drug-resistant *A. baumannii*, as this domain contributes to stabilization of cell wall integrity and antimicrobial resistance [79, 81]. In *E. coli*, EcOmpA competes with IgaA for binding to RcsF, and the OmpA-like domain of EcOmpA controls the regulator of capsule synthesis (Rcs) system [159, 160]. The Rcs system senses envelope damage and regulates genes associated with bacterial survival and virulence. Upon stress signaling, RcsF interacts with IgaA, leading to de-repression of the phosphorelay and activation of Rcs cascades. Homologs of RcsF are predicted in the genome of *A. baumannii*, and its predicted three-dimensional structure resembles that of RcsF in *E. coli* (Unpublished data). Inhibitors that bind to the OmpA-like domain of AbOmpA can block cell wall integration and efflux pump activity, and activate uncontrolled signaling of the Rcs system. Moreover, a nuclear localization signal within the OmpA-like domain of AbOmpA has been identified, facilitating its targeting to the nuclei of host cells [69]. Inhibitors targeting β-barrel and the C-terminal globular domain of AbOmpA may neutralize the cytotoxic activity of OMV-associated AbOmpA. However, these domains are located within the vesicular membrane and lumen, which may pose challenge for the penetration of neutralizing agents.

Although AbOmpA has emerged as a promising therapeutic target against drug-resistant *A. baumannii*, several critical challenges must be addressed before it can be translated into clinical applications. One major concern is the potential for target saturation due to the release of OMVs containing AbOmpA, which may sequester therapeutic agents and reduce their efficacy at the bacterial surface [161]. While interaction with OMV-associated AbOmpA might reduce virulence, the competitive binding of AbOmpA inhibitors to OMV-associated AbOmpA could compromise their effectiveness against outer membrane-integrated AbOmpA. Furthermore, access to AbOmpA may be hindered by surface structures such as the polysaccharide capsule and LOS, limiting the binding and activity of potential therapeutics [161]. Immune evasion mechanisms, including antigenic variation and surface modifications, further complicate the development of AbOmpA-targeted therapies, potentially compromising their long-term efficacy [162]. Although anti-virulence agents exert low selective pressure, the potential for resistance to small molecules or peptides targeting AbOmpA should be considered. Additionally, AbOmpA-targeting drugs may need to be used in combination with bactericidal agents, as anti-virulence drugs alone cannot directly kill *A. baumannii* and the patient’s immune system may struggle to eradicate invading bacteria. Overcoming these multifaceted barriers will be essential to advance AbOmpA-targeted strategies toward successful clinical implementation.

## Conclusions

Significant advancements have been made in understanding AbOmpA-mediated virulence over the past 2 decades, establishing AbOmpA as a promising therapeutic target for combating drug-resistant *A. baumannii*. Notable progress has been achieved in the development of AbOmpA-targeted therapeutics for *A. baumannii* infections. Although several potential molecules that inhibit *AbompA* expression of and neutralize AbOmpA have been shown to significantly reduce *A. baumannii* virulence both in vitro and in vivo, none have advanced to clinical trials. Moreover, the AbOmpA-targeted therapeutic approach does not account for the release of OMVs from *A. baumannii* and the presence of AbOmpA in these vesicles. Given that OMVs and OMV-associated AbOmpA contribute to virulence, antimicrobial resistance, and immunopathology, their role in AbOmpA-targeted therapies should be carefully considered. However, research on OMV biogenesis, the packaging of AbOmpA in OMVs, and the stability of AbOmpA within the vesicular membrane remains limited. Further investigations are necessary to elucidate the role of AbOmpA in the pathogenesis of *A. baumannii* and the clinical potential of AbOmpA-targeted therapeutics. Addressing these knowledge gaps will be crucial for advancing AbOmpA-based interventions to combat *A. baumannii* infections.

## Data Availability

Not applicable.

## Abbreviations

AbOmpA: Outer membrane protein A of *Acinetobacter baumannii*
OMV: Outer membrane vesicle
MDR: Multidrug-resistance
CRAB: Carbapenem-resistant *Acinetobacter baumannii*
RND: Resistance-modulation-division
LOS: Lipooligosaccharides
EcOmpA: *E. coli* OmpA
IL: Interleukin
H-NS: Histone-like nucleoid structuring
rAbOmpA: Recombinant AbOmpA
mTOR: Mammalian target of rapamycin
MAPK: Mitogen-activated protein kinases
DC: Dendritic cell
MHC: Major histocompatibility complex
TNF: Tumor necrosis factor
PI3K: Phosphoinositide 3-kinase
MICs: Minimum inhibitory concentrations
AnOmpA: OmpA of* A. nosocomialis*
EVs: Extracellular vesicles
ZrlA: Zinc uptake regulator-regulated lipoprotein A
VDAC: Voltage-dependent anion channel
ROS: Reactive oxygen species
DRP1: Dynamin‑related protein 1
TLR2: Toll-like receptor 2
NLRP3: NOD-like receptor protein 3
PAMPs: Pathogen-associated molecular patterns
Rcs: Regulator of capsule synthesis
